# Supplementary material for: Costatone C—A New Halogenated Monoterpene from the New Zealand Red Alga Plocamium angustum
Source: Mar Drugs. 2019 Jul 19;17(7):418. doi: 10.3390/md17070418 (PMC6669586; doi:10.3390/md17070418)

marine drugs

Article

Costatone C – a New Halogenated Monoterpene from the New Zealand Red Alga "*Plocamium angustum*"

Joe Bracegirdle [1,2,4], Zaineb Sohail [5] Michael J. Fairhurst [2,3], Monica L. Gerth [2,3,4], Giuseppe C. Zuccarello [3], Muhammad Ali Hashmi [6], Robert A. Keyzers [1,2,4,*]

1 School of Chemical and Physical Sciences, Victoria University of Wellington, Wellington 6012, New Zealand; joe.bracegirdle@vuw.ac.nz (J.B.)
2 Centre for Biodiscovery, Victoria University of Wellington, Wellington 6012, New Zealand.
3 School of Biological Sciences, Victoria University of Wellington, Wellington 6012, New Zealand; mike.fairhurst@vuw.ac.nz (M.J.F.); monica.gerth@vuw.ac.nz (M.L.G.); joe.zuccarello@vuw.ac.nz (G.C.Z.)
4 Maurice Wilkins Centre for Molecular Biodiscovery, New Zealand.
5 Department of Life Sciences, University of Management and Technology, C-II, Johar Town Lahore 54770, Pakistan; z.sohail.y@gmail.com (Z.S.)
6 Department of Chemistry, University of Education, Attock Campus, Attock 43600, Pakistan; i4hashmi@hotmail.com (M.A.H.)
* Correspondance: robert.keyzers@vuw.ac.nz (R.A.K); Tel.: +64 4 4635117

Table S1: NMR data (600 MHz for ^{1}H, 150 MHz for ^{13}C, CD_3OD) of **7** 3

Figure S1: ^{1}H Spectrum (600 MHz, CD_3OD) of **7** 3

Figure S2: ^{13}C Spectrum (150 MHz, CD_3OD) of **7** 4

Figure S3: COSY Spectrum (600 MHz, CD_3OD) of **7** 4

Figure S4: HSQC Spectrum (600 MHz, CD_3OD) of **7** 5

Figure S5: HMBC Spectrum (600 MHz, CD_3OD) of **7** 5

Figure S6: ROESY Spectrum (600 MHz, CD_3OD) of **7** 6

Table S2: NMR data (600 MHz for ^{1}H, 150 MHz for ^{13}C, $CDCl_3$) of **7** 6

Figure S7: ^{1}H Spectrum (600 MHz, $CDCl_3$) of **7** 7

Figure S8: ^{13}C Spectrum (150 MHz, $CDCl_3$) of **7** 7

Figure S9: COSY Spectrum (600 MHz, $CDCl_3$) of **7** 8

Figure S10: HSQC Spectrum (600 MHz, $CDCl_3$) of **7** 8

Figure S11: HMBC Spectrum (600 MHz, $CDCl_3$) of **7** 9

Figure S12: NOESY Spectrum (600 MHz, $CDCl_3$) of **7** 9

Figure S13: (-)-HR-APCI-MS Spectrum of **7** 10

Figure S14: IR Spectrum (film) of **7** 10

Figure S15: ^{1}H Spectrum (600 MHz, $CDCl_3$) of **8** **11**

Figure S16: ^{13}C Spectrum (150 MHz, $CDCl_3$) of **8** **11**

Figure S17: ^{1}H Spectrum (600 MHz, $CDCl_3$) of *S*-MTPA ester of **8** (**12a**) **12**

Figure S18: COSY Spectrum (600 MHz, $CDCl_3$) of *S*-MTPA ester of **8** (**12a**) **12**

Figure S19: ^{1}H Spectrum (600 MHz, $CDCl_3$) of *R*-MTPA ester of **8** (**12b**) **13**

Figure S20: COSY Spectrum (600 MHz, $CDCl_3$) of *R*-MTPA ester of **8** (**12b**) **13**

Figure S21: Antibacterial bioassay results for **7** and **8** **14**

Figure S22: Phylogenetic analysis of *Plocamium* species based on COI data **15**

Figure S23: Unscaled and scale ECD spectra **16**

Figure S24: Relative energies of all computed conformers of costatone C (**7**). **17**

Table S1 – NMR data (600 MHz for ^{1}H, 150 MHz for ^{13}C, CD_3OD) of **7**

position	^{13}C (δ)	^{1}H (δ, mult., *J in Hz)*	COSY	HMBC
1	117.5	6.28 (quin, 1.4)	3, 9	2, 3, 9
2	138.5			
3	73.8	4.22 (dd, 11.7, 2.6)	1, 4, 9	1, 2, 4, 5, 7, 9
4	39.1	2.45 (dt, 12.9, 11.9)	3, 4, 5	2, 3, 5
		2.15 (ddd, 12.9, 4.4, 2.7)	3, 4, 5	3, 5
5	54.5	4.75 (dd, 12.0, 4.4)	4	3, 4, 6, 10
6	73.6			
7	83.4	4.29 (dd, 11.7, 3.9)	8	3, 6, 5, 8, 10
8	29.0	3.94 (t, 11.6)	7, 8	6, 7
		3.73 (dd, 11.6, 3.9)	7, 8	7
9	13.1	1.84 (d, 1.4)	1, 3	1, 2, 3
10	28.9	1.70 (s)		5, 6, 7

Figure S1 – ^{1}H Spectrum (600 MHz, CD_3OD) of **7**

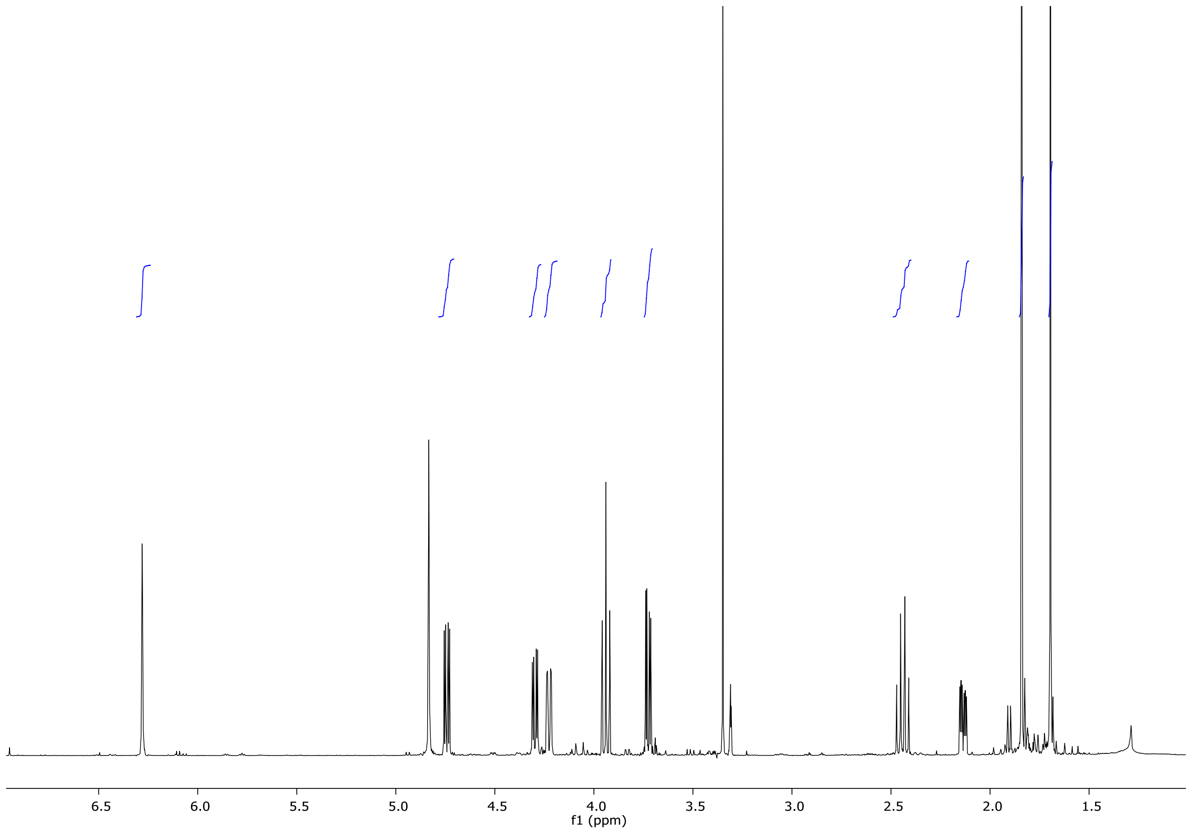

Figure S2 – ^{13}C Spectrum (150 MHz, CD_3OD) of **7**

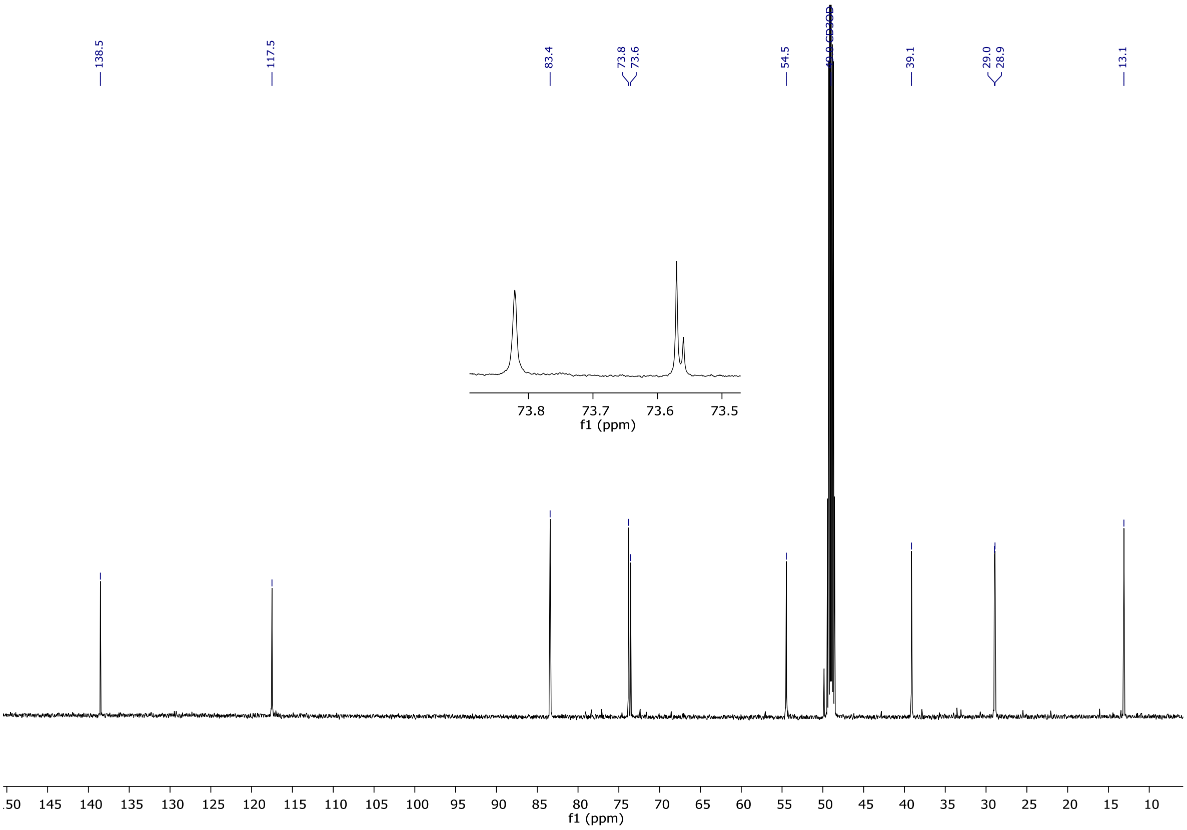

Figure S3 – COSY Spectrum (600 MHz, CD_3OD) of **7**

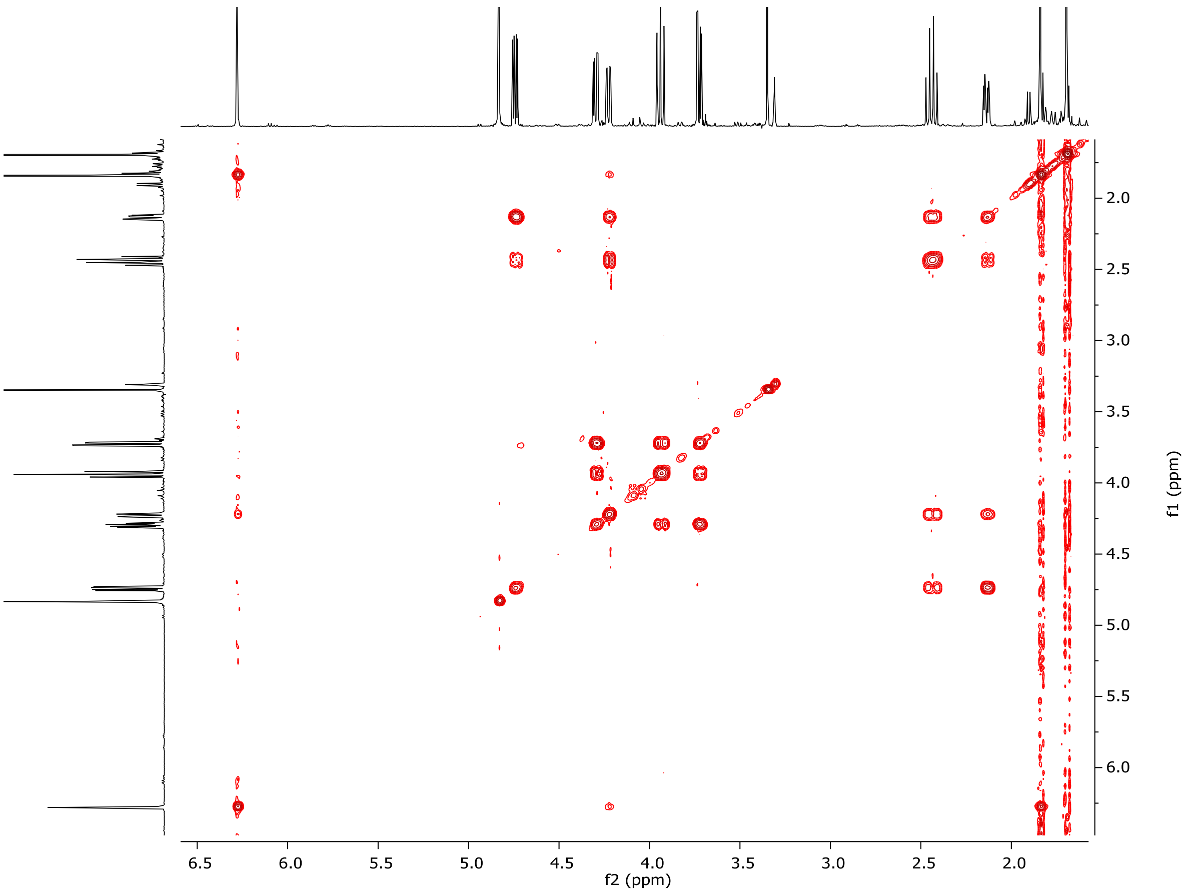

Figure S4 – HSQC Spectrum (600 MHz, CD_3OD) of **7**

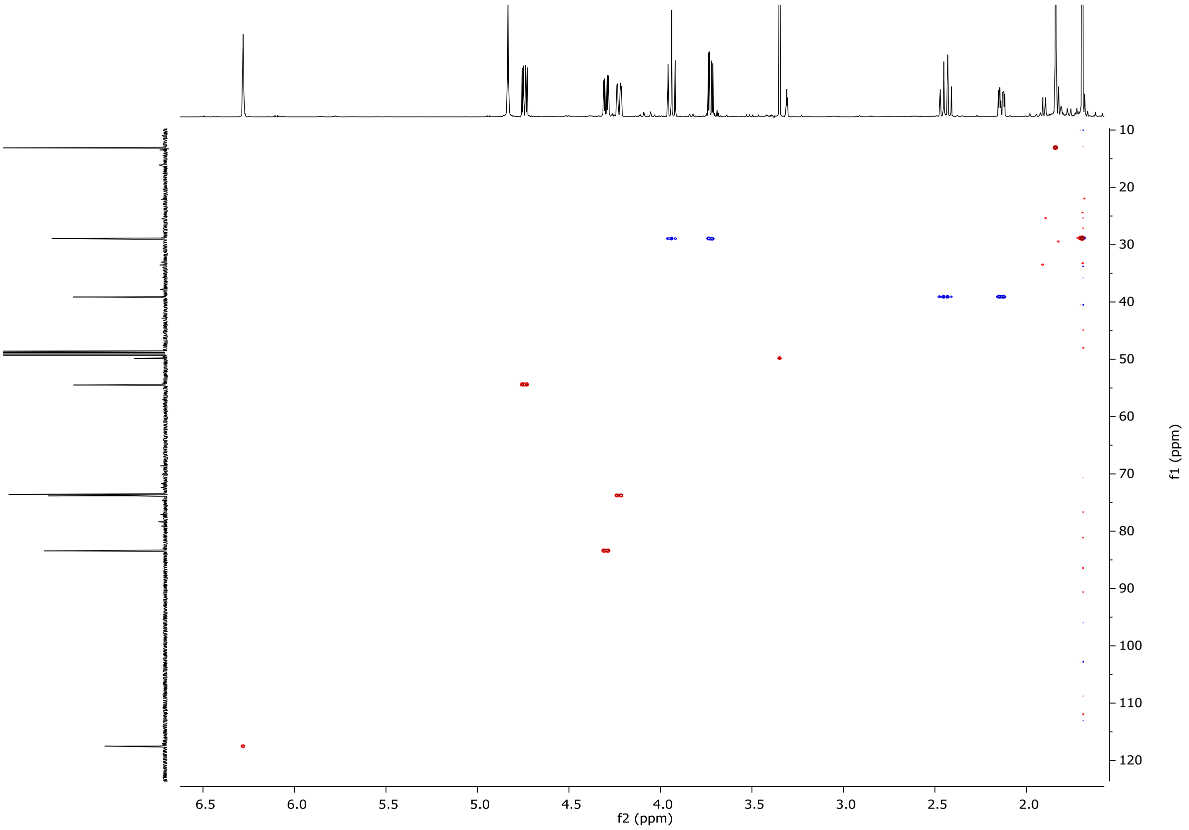

Figure S5 – HMBC Spectrum (600 MHz, CD_3OD) of **7**

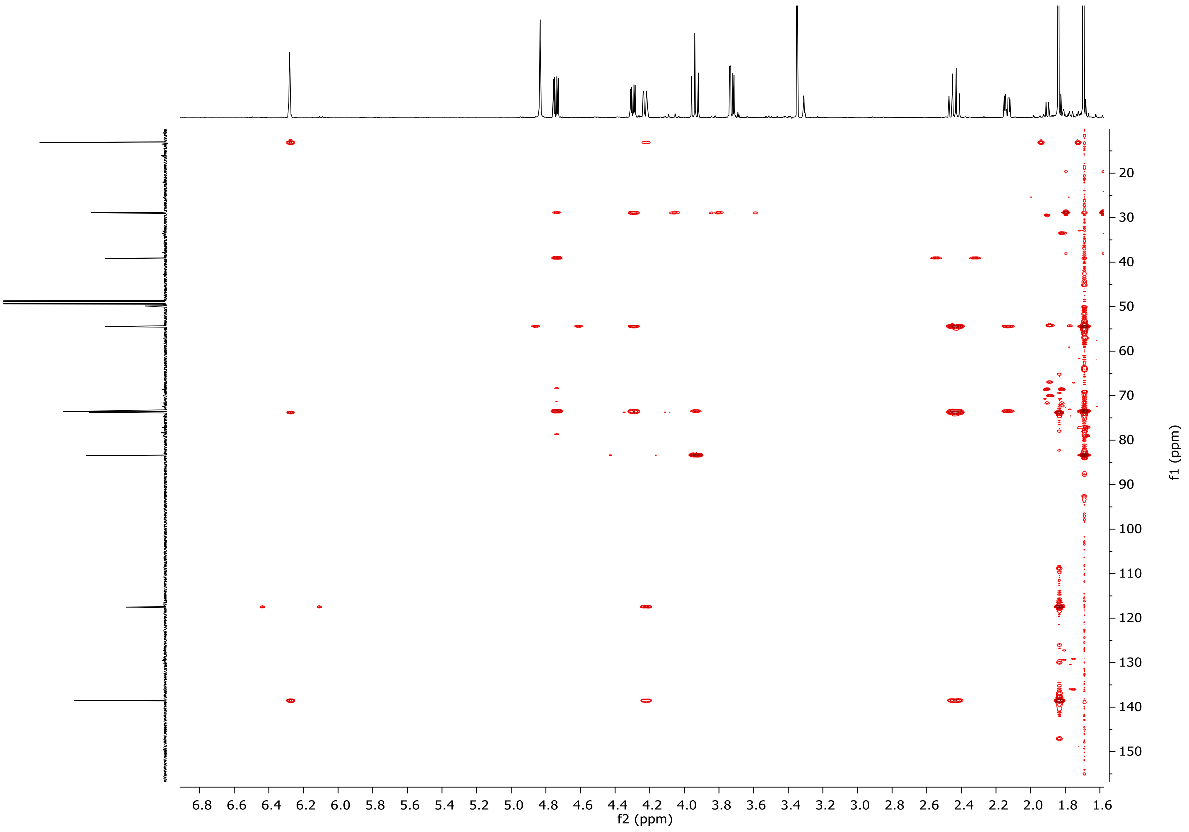

Figure S6 – ROESY Spectrum (600 MHz, CD_3OD) of **7**

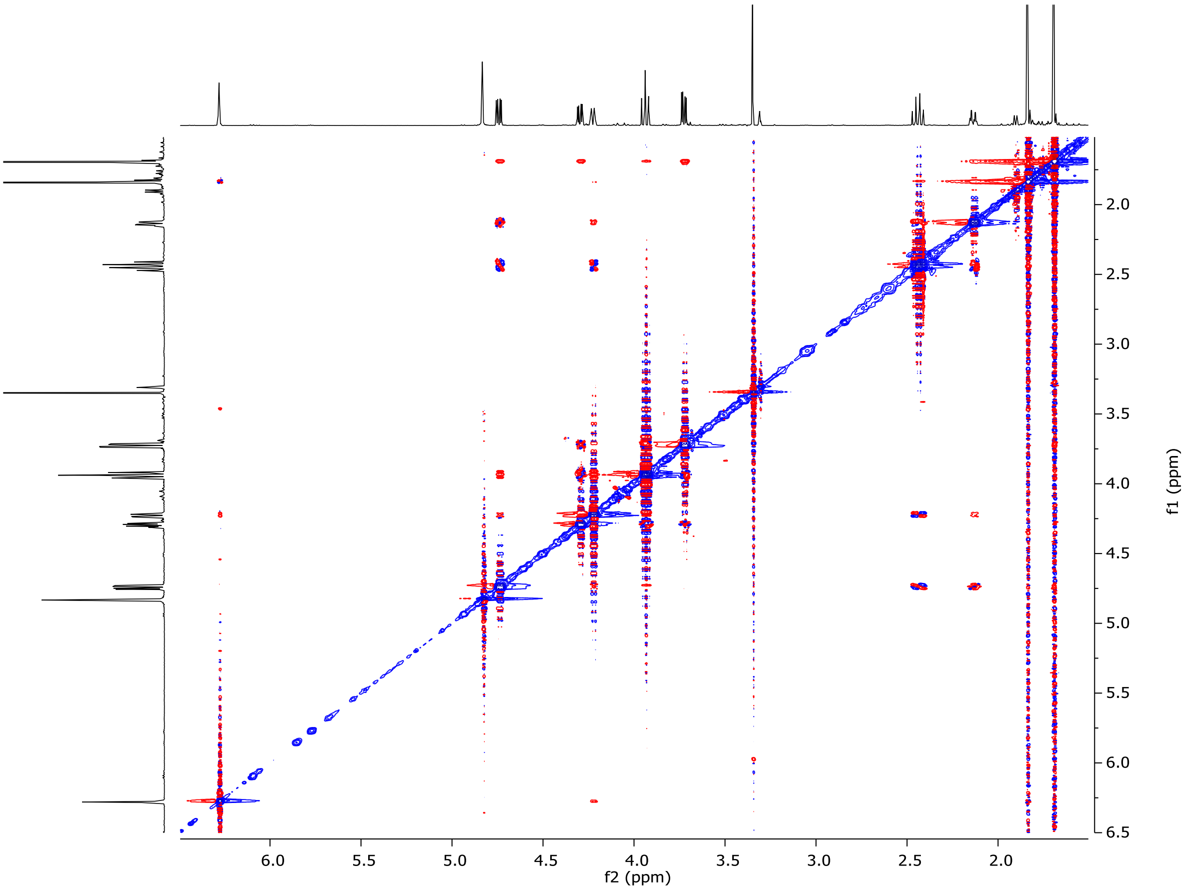

Table S2 – NMR data (600 MHz for ^{1}H, 150 MHz for ^{13}C, $CDCl_3$) of **7**

position	^{13}C (δ)	^{1}H (δ, mult., *J in Hz)*	COSY	HMBC
1	117.3	6.25 (quin, 1.4)	3, 9	2, 3, 9
2	136.7			
3	73.2	4.12 (dd, 11.7, 2.8)	1, 4, 9	1, 2, 5, 9
4	37.7	2.56 (dt, 13.3, 11.9)	3, 4, 5	2, 3, 5, 6
		2.16 (ddd, 13.3, 4.4, 2.8)	3, 4, 5	5, 6
5	53.1	4.37 (m, overlap)	4	3, 4, 6, 10
6	71.5			
7	81.7	4.37 (m, overlap)	8	3, 6, 5, 8, 10
8	27.8	3.61 (m)	7	6, 7
9	13.3	1.87 (d, 1.4)	1, 3	1, 2, 3
10	28.6	1.72 (s)		5, 6, 7

Figure S7 – [1]H Spectrum (600 MHz, $CDCl_3$) of **7**

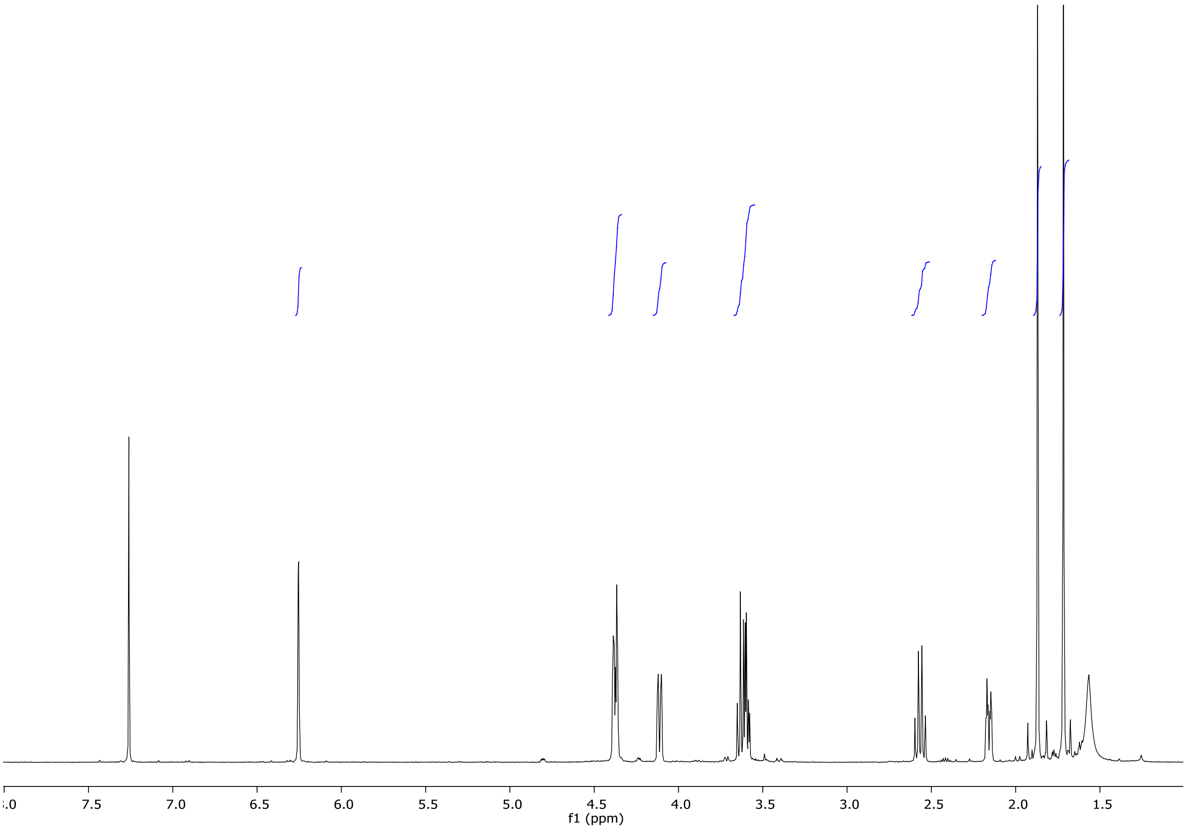

Figure S8 – [13]C Spectrum (150 MHz, $CDCl_3$) of **7**

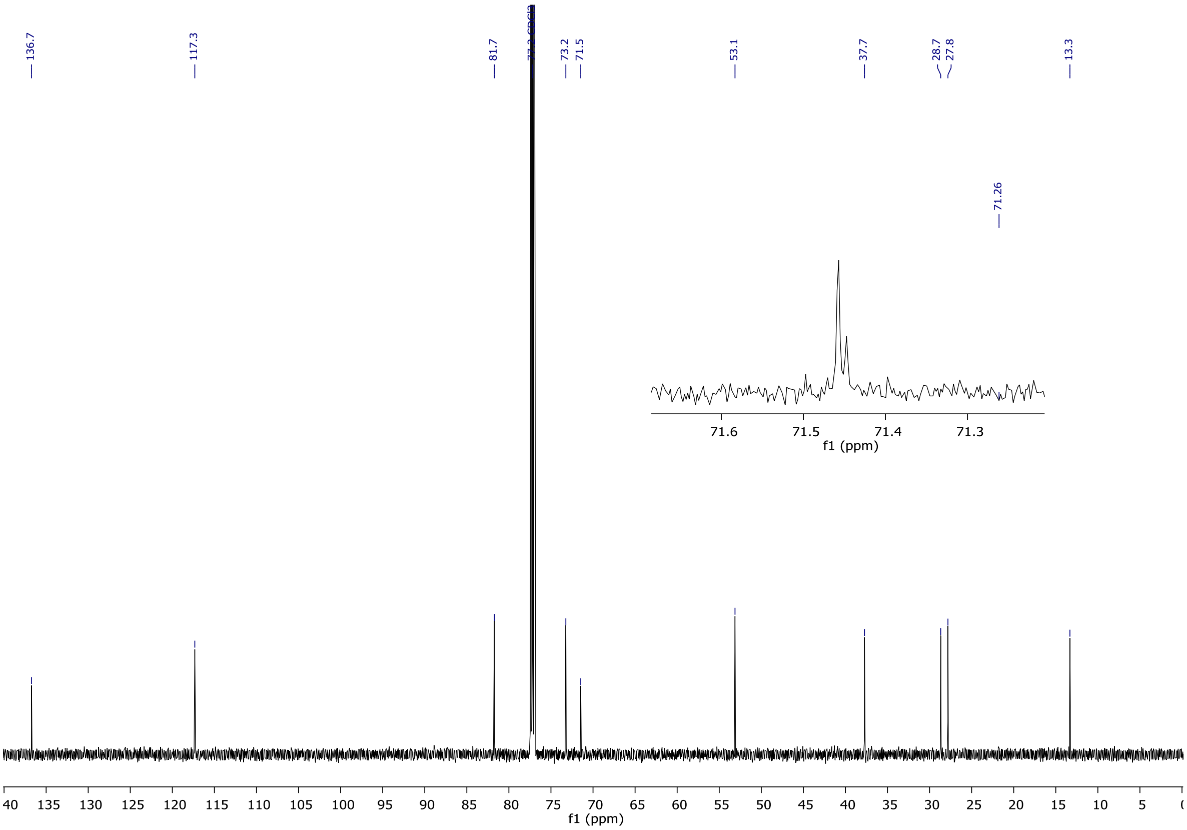

Figure S9 – COSY Spectrum (600 MHz, $CDCl_3$) of **7**

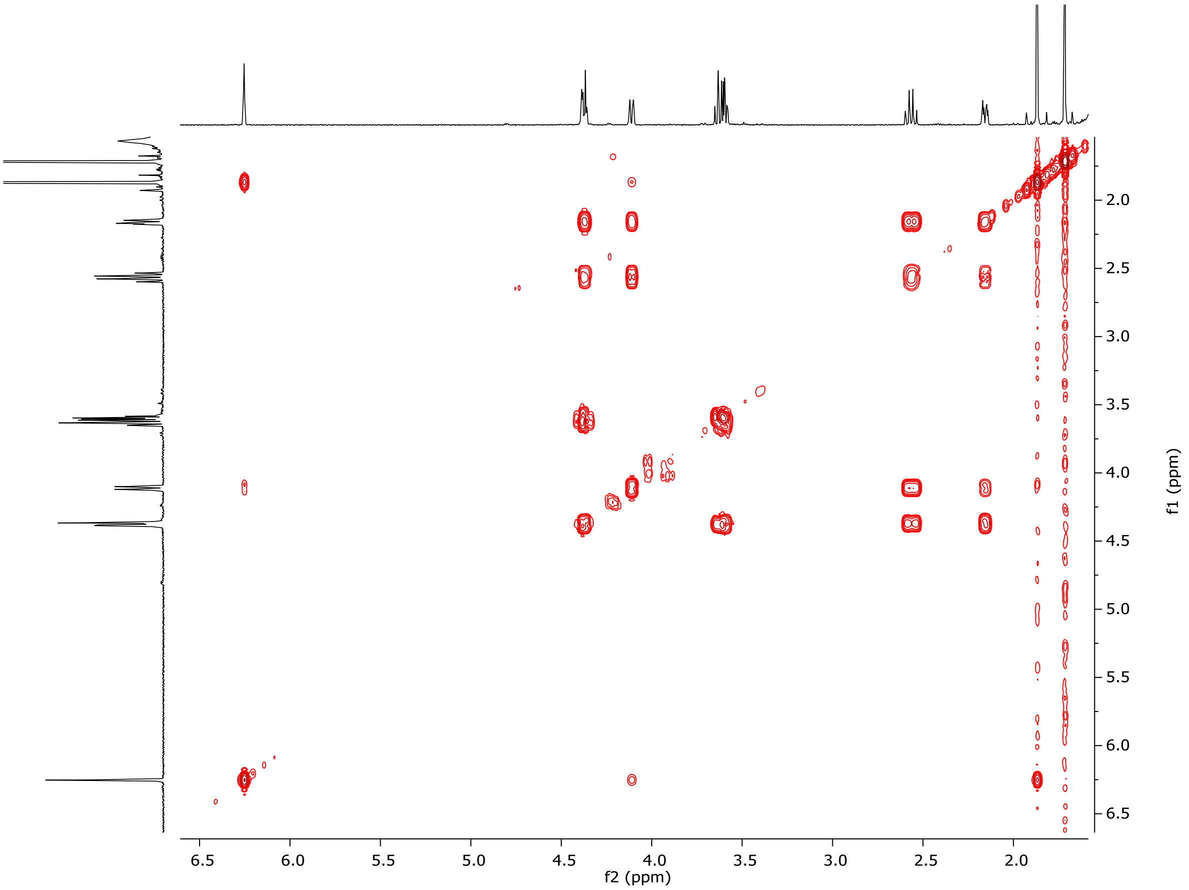

Figure S10 – HSQC Spectrum (600 MHz, $CDCl_3$) of **7**

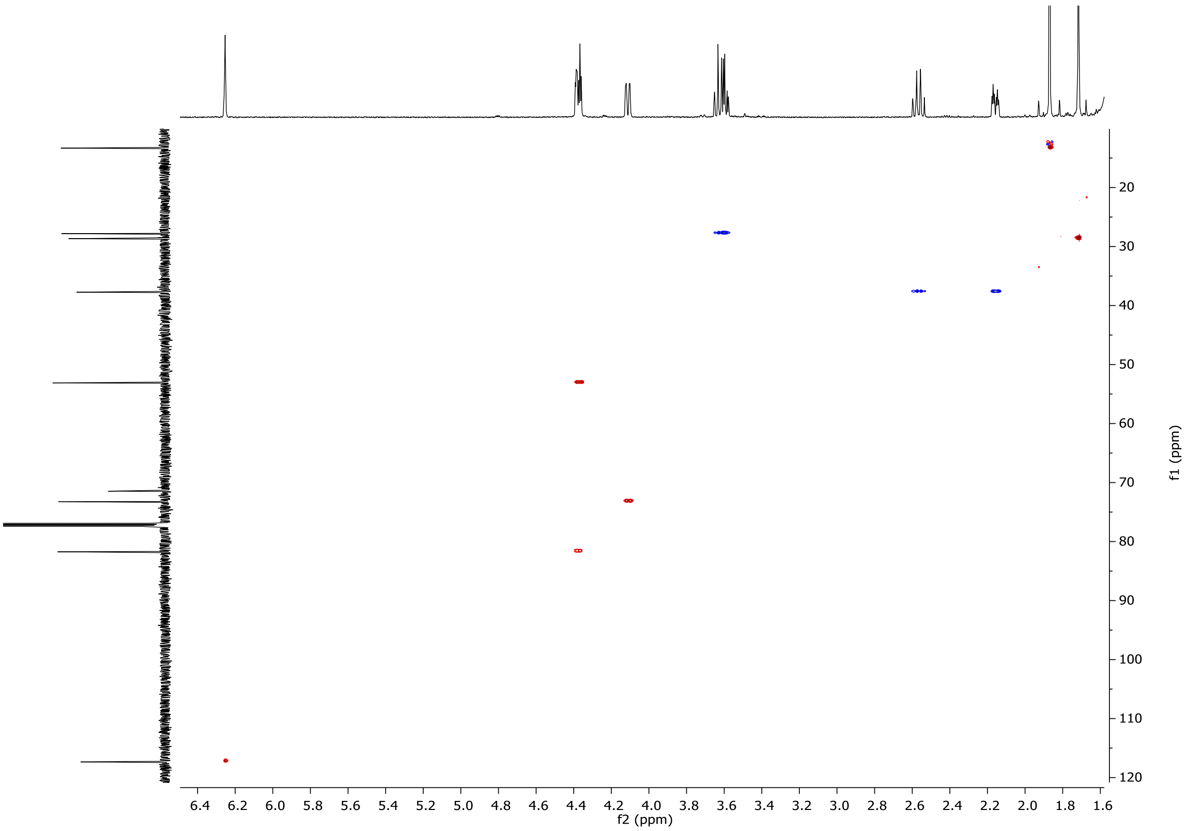

Figure S11 – HMBC Spectrum (600 MHz, $CDCl_3$) of **7**

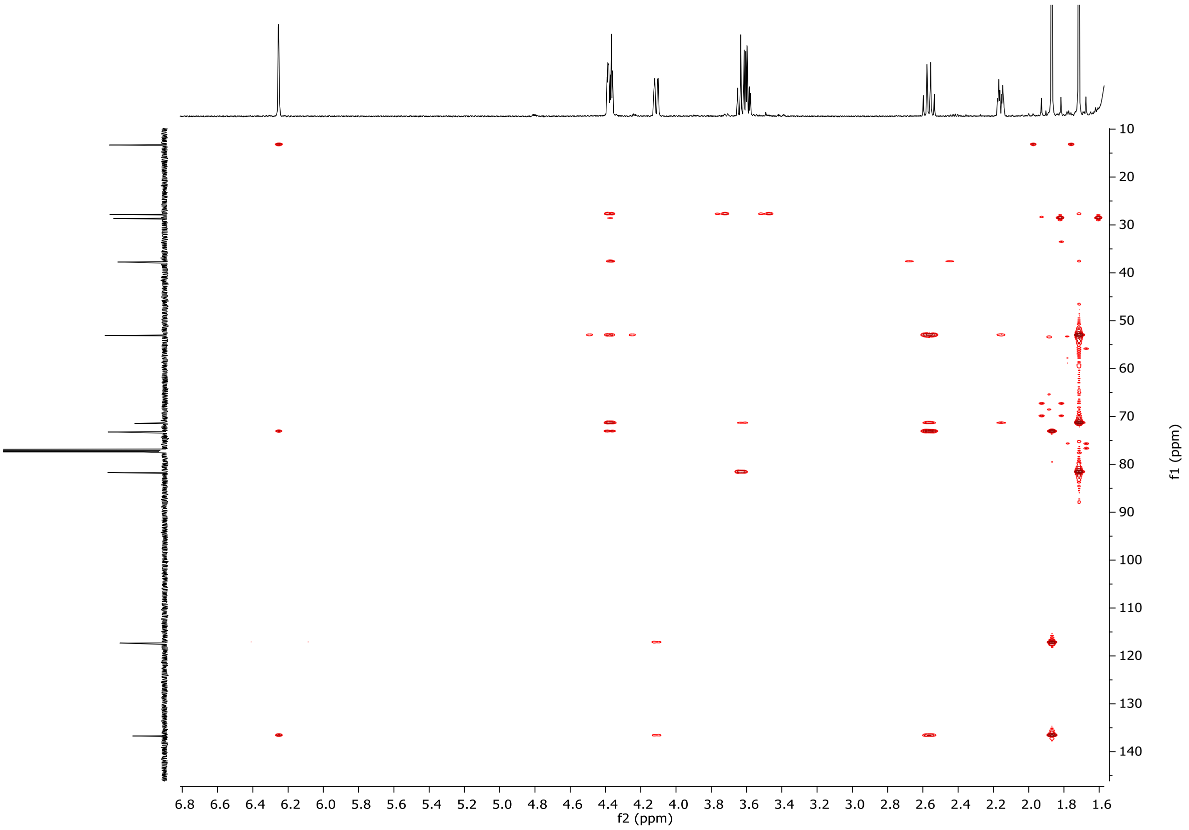

Figure S12 – NOESY Spectrum (600 MHz, $CDCl_3$) of **7**

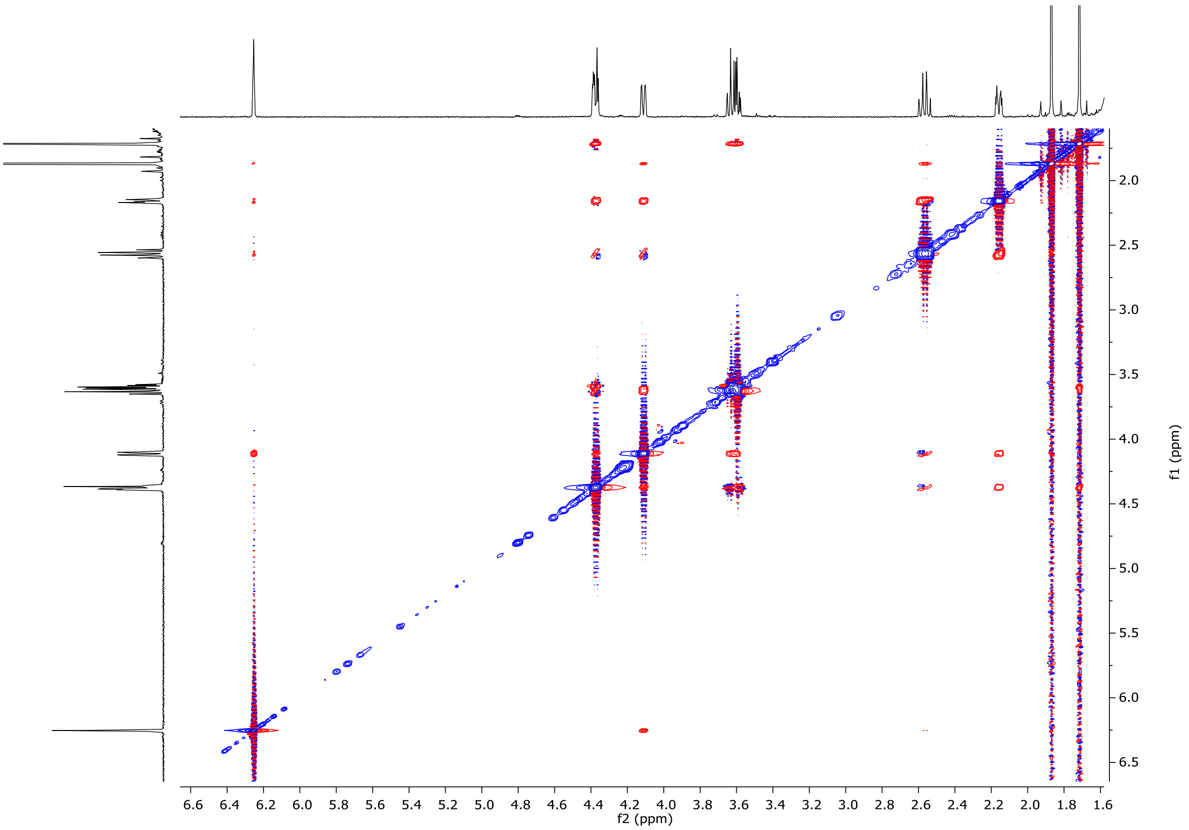

Figure S13 – (-)HR-APCI-MS Spectrum of **7**

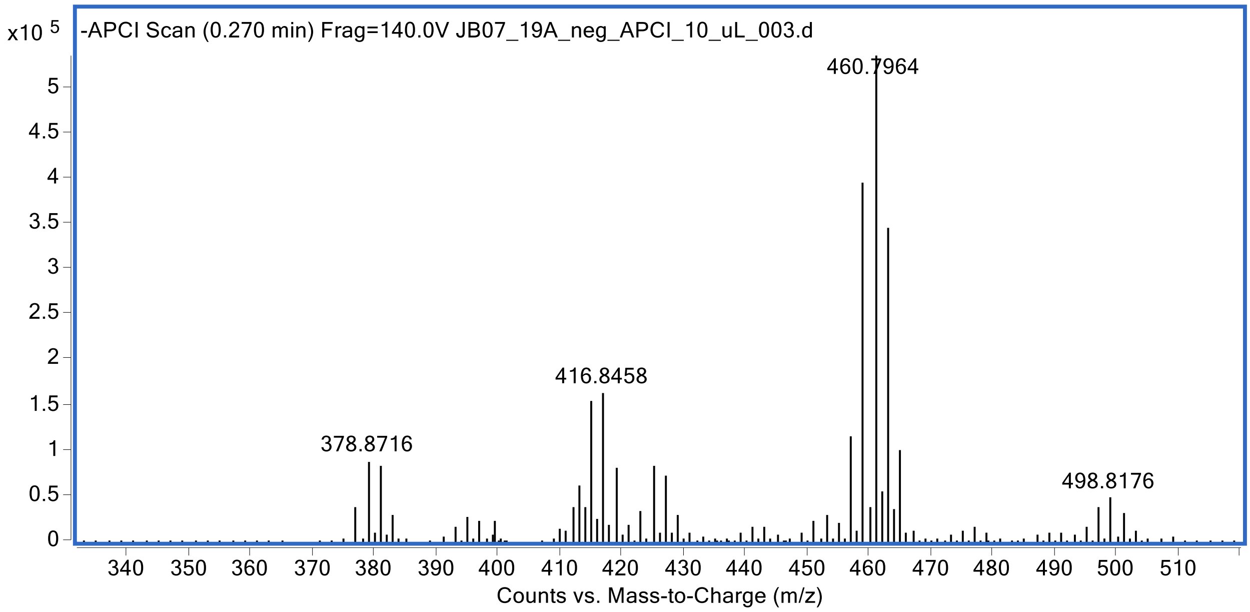

Figure S14 – IR Spectrum (film) of **7**

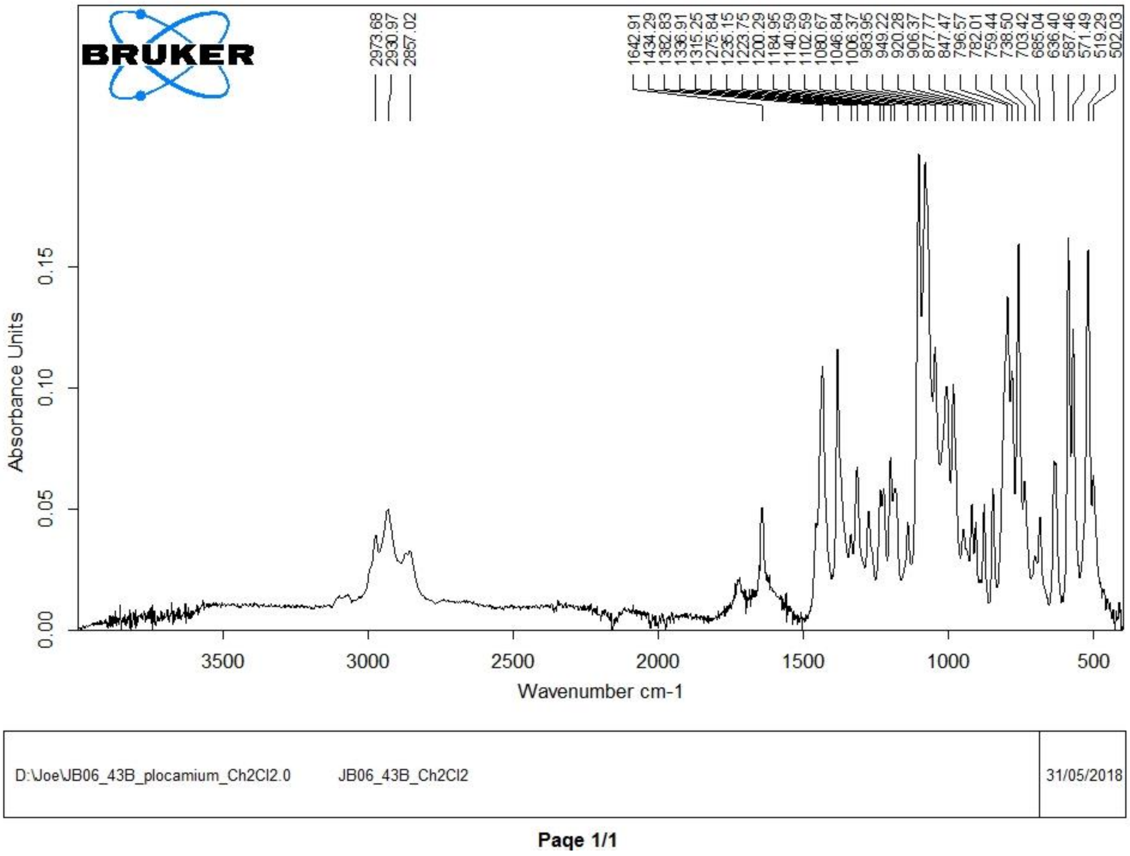

Figure S15 – ^{1}H Spectrum (600 MHz, $CDCl_3$) of **8**

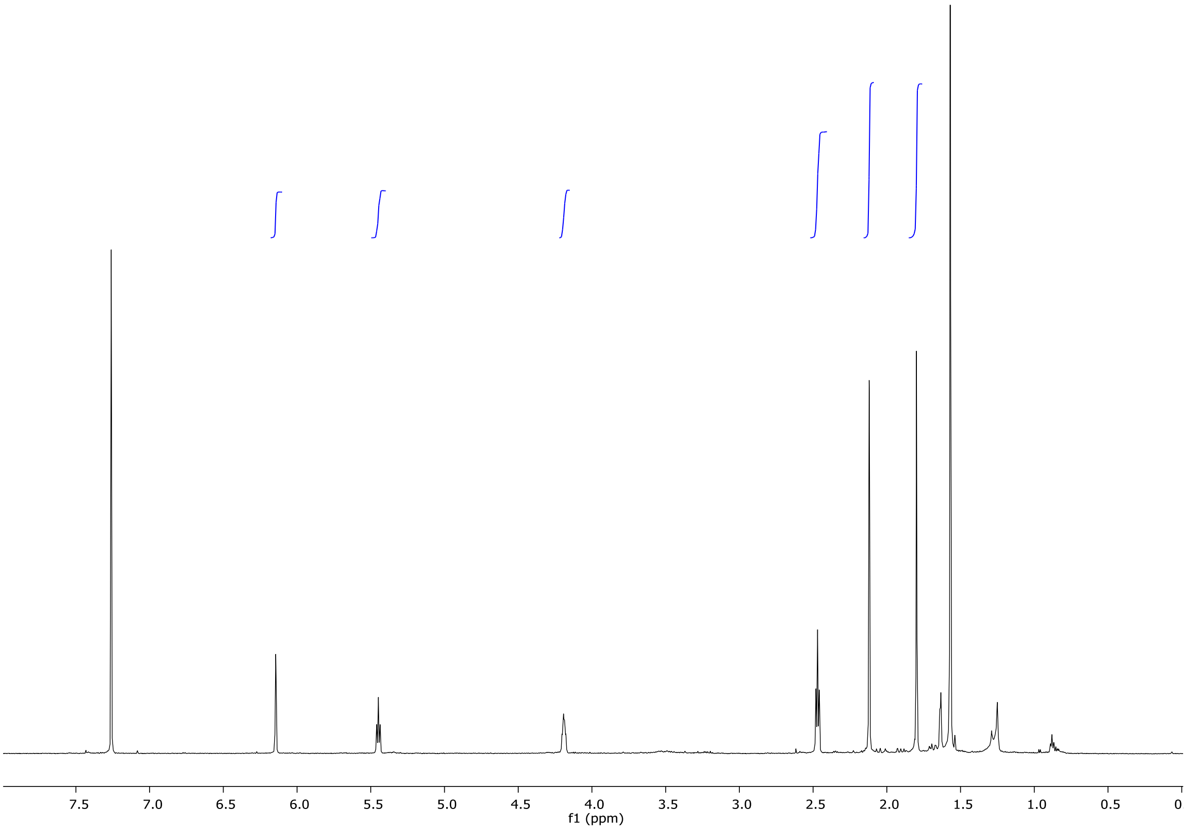

Figure S16 – ^{13}C Spectrum (150 MHz, $CDCl_3$) of **8**

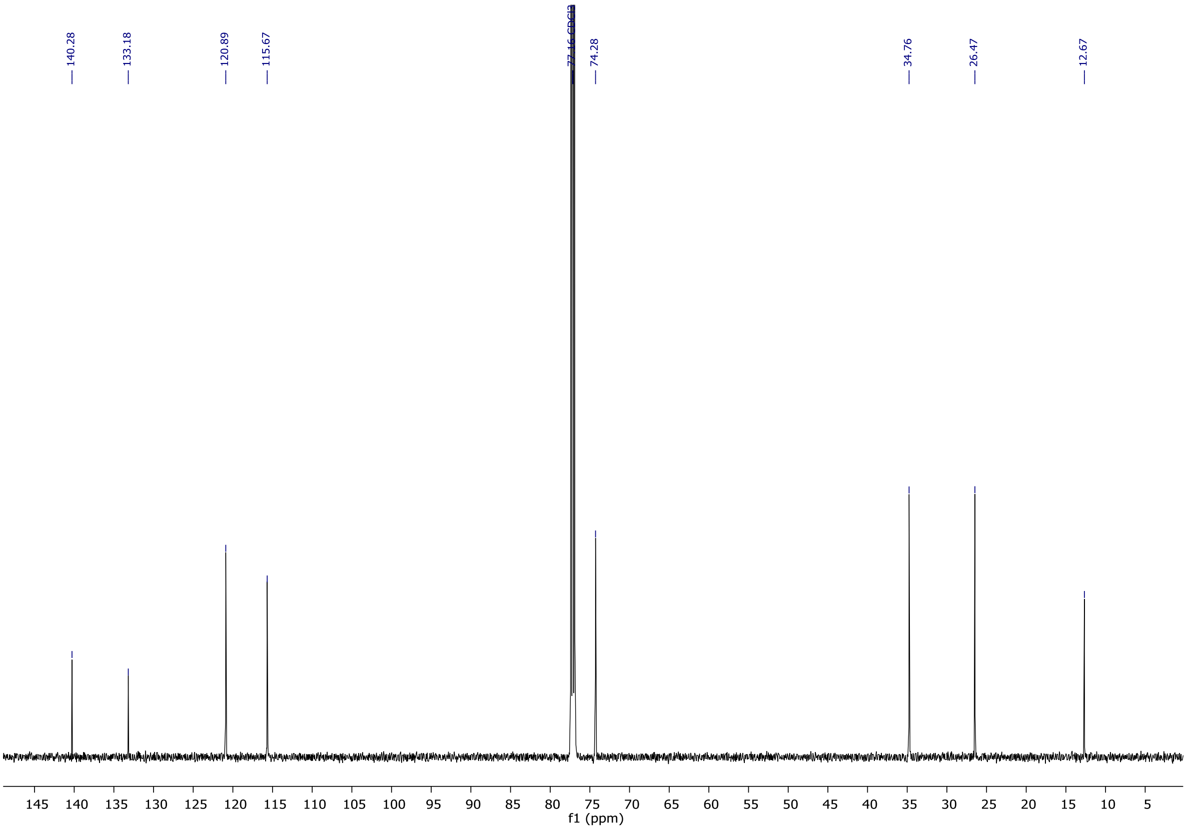

Figure S17 – ^{1}H Spectrum (600 MHz, $CDCl_3$) of *S*-MTPA ester of **8** (**12a**)

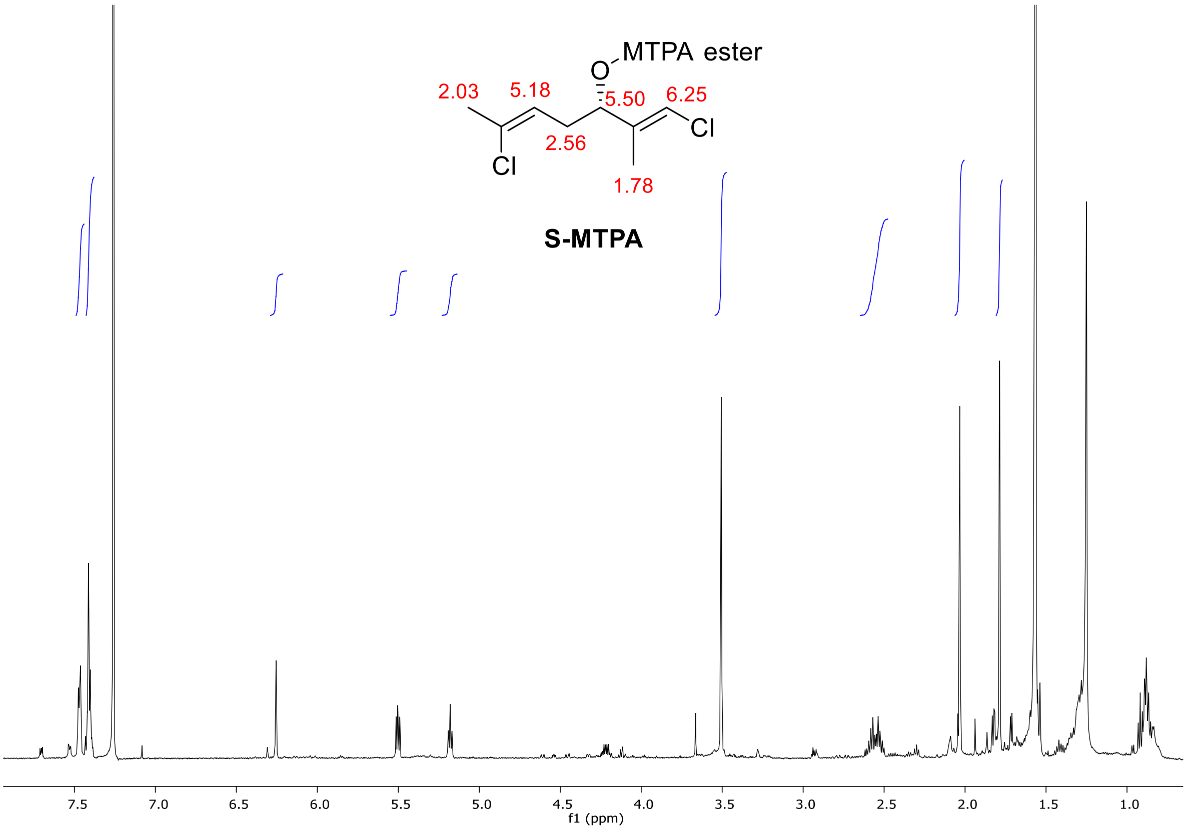

Figure S18 – COSY Spectrum (600 MHz, $CDCl_3$) of *S*-MTPA ester of **8** (**12a**)

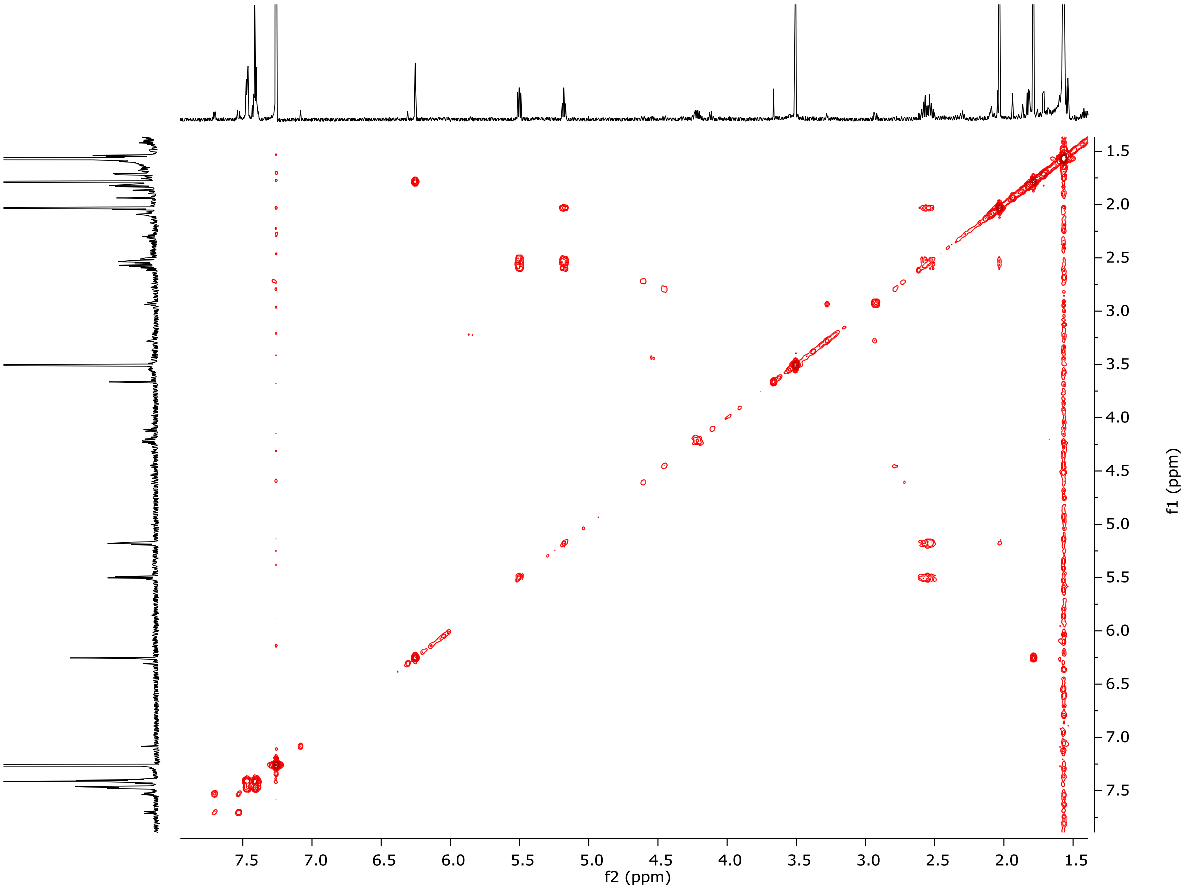

Figure S19 – [1]H Spectrum (600 MHz, $CDCl_3$) of *R*-MTPA ester of **8** (**12b**)

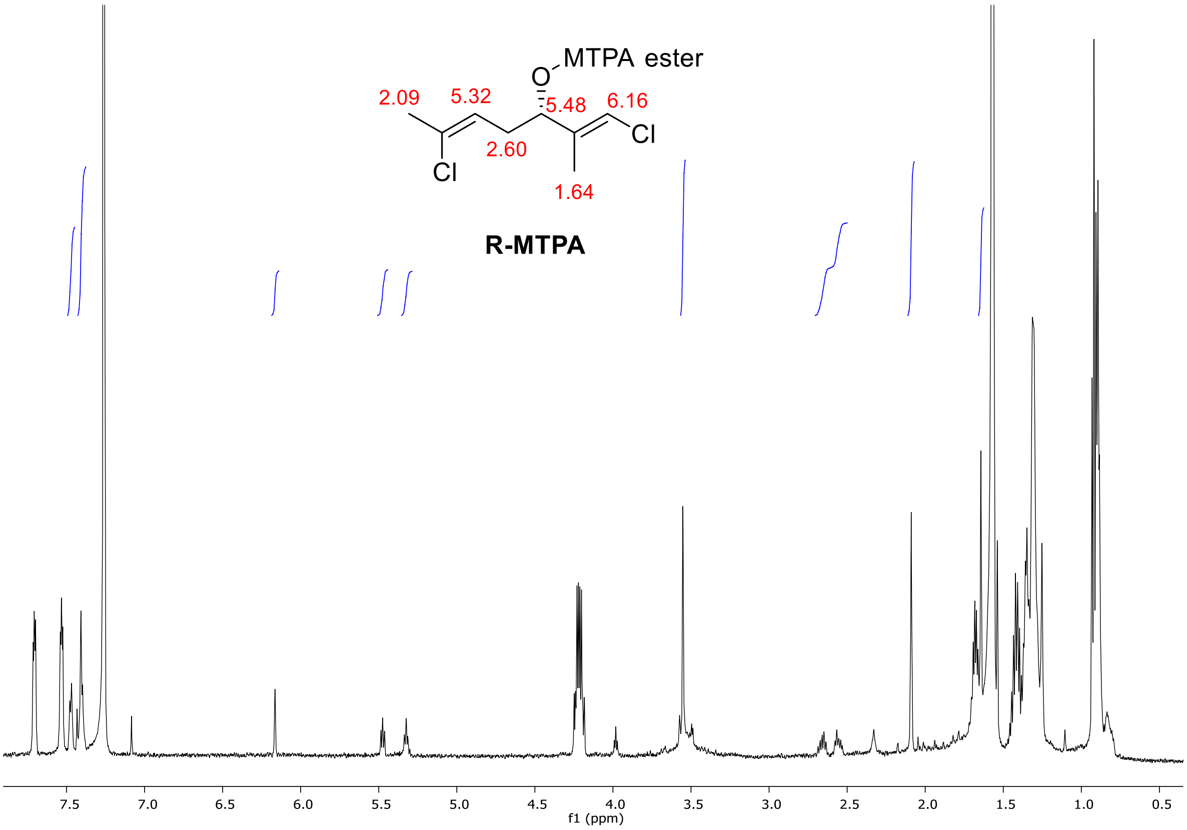

Figure S20 – COSY Spectrum (600 MHz, $CDCl_3$) of *R*-MTPA ester of **8** (**12b**)

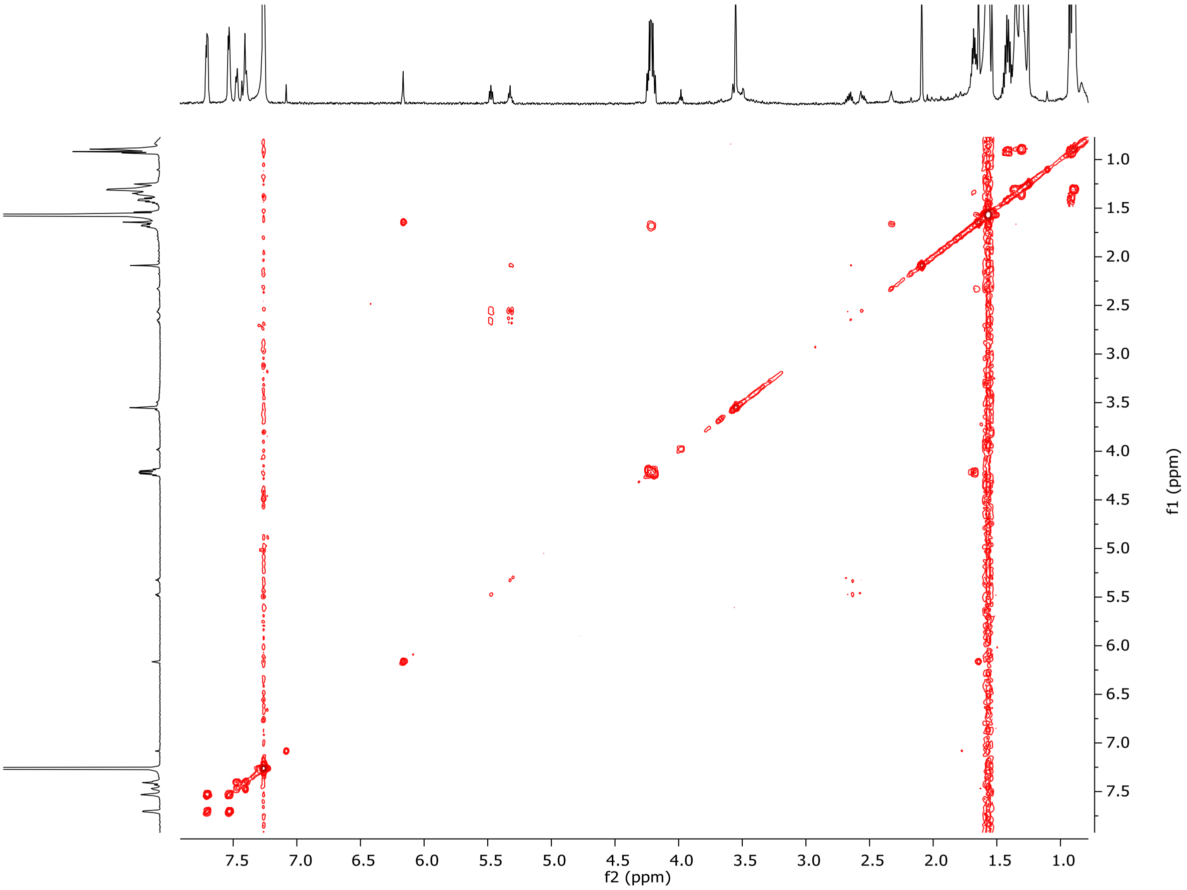

Figure S21 – Antibacterial testing of **7** and **8**. (A) Initial screening for growth inhibition of *P. aeruginosa* (black circles) and *Staphylococcus aureus* (white triangles). Cntl: 1% DMSO control. The individual data points are shown; horizontal lines represents the mean of three replicates. (B) Determination of the minimum inhibitory concentration of **7** towards *S. aureus* (grey bars) and *S. epidermidis* (black bars). The means and standard deviations of three replicates are shown as bars and lines, respectively.

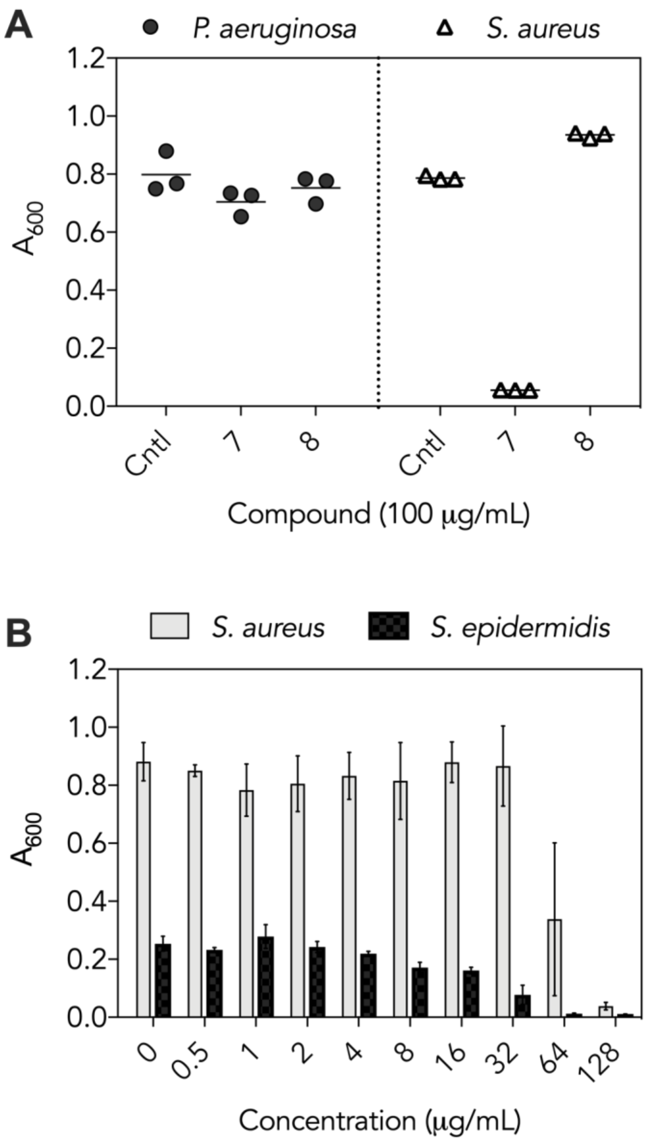

Figure S22 – Phylogenetic analysis (maximum-likelihood) of *Plocamium* species based on COI data. Bootstrap values >50% on branches shown. Scale bar = substitutions/site

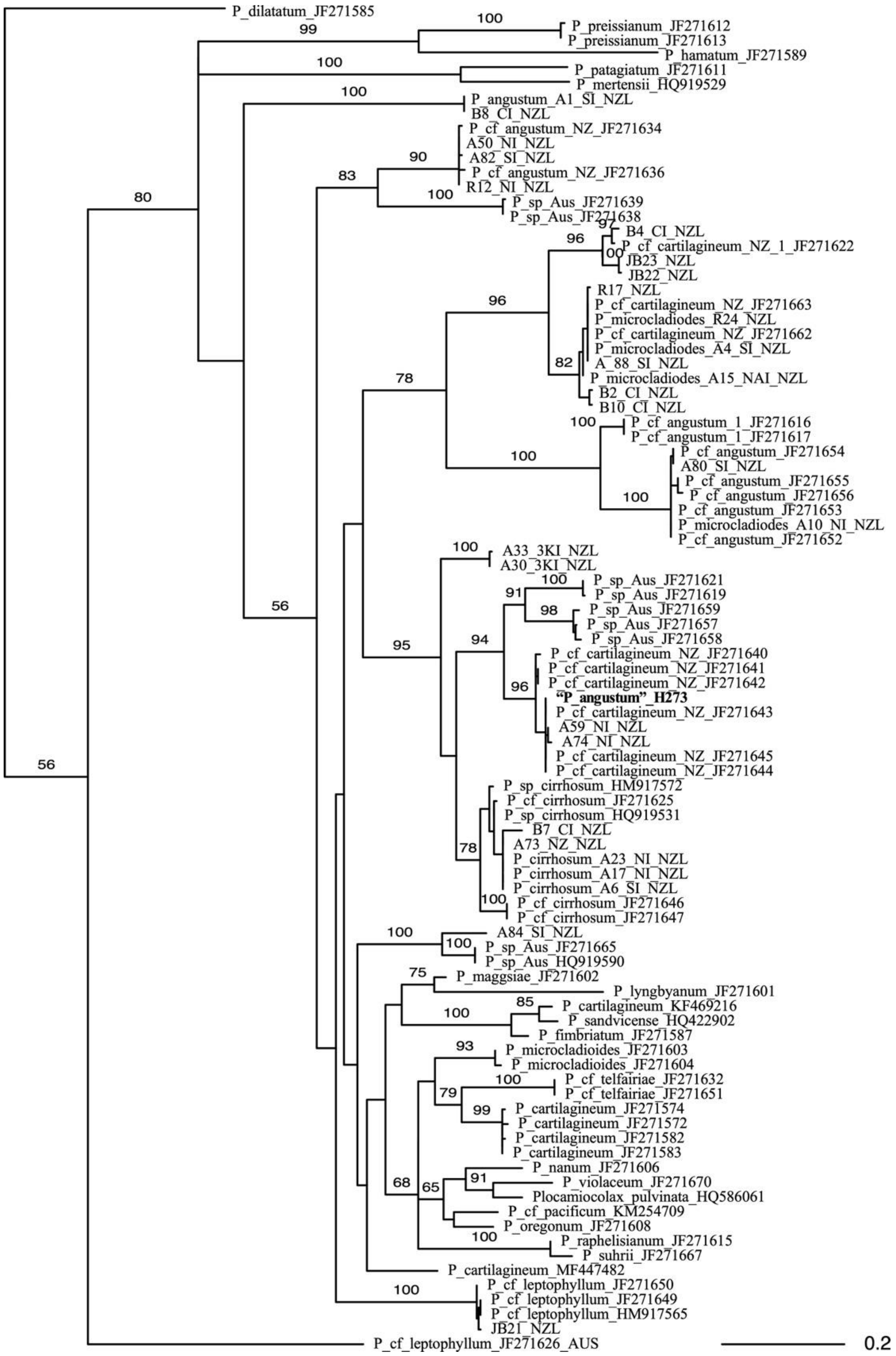

Figure S23 - Comparison of experimental ECD spectrum with calculated unscaled, calculated with only intensity scaling, and calculated with both intensity and frequency scaling (Figure 3 in the manuscript). Due to the much lower intensity of the calculated values, the values were multiplied by 20. The frequency was shifted 7 nm to the left for the calculated spectrum to compare with the experimental spectrum whose data was available for 190-280 nm.

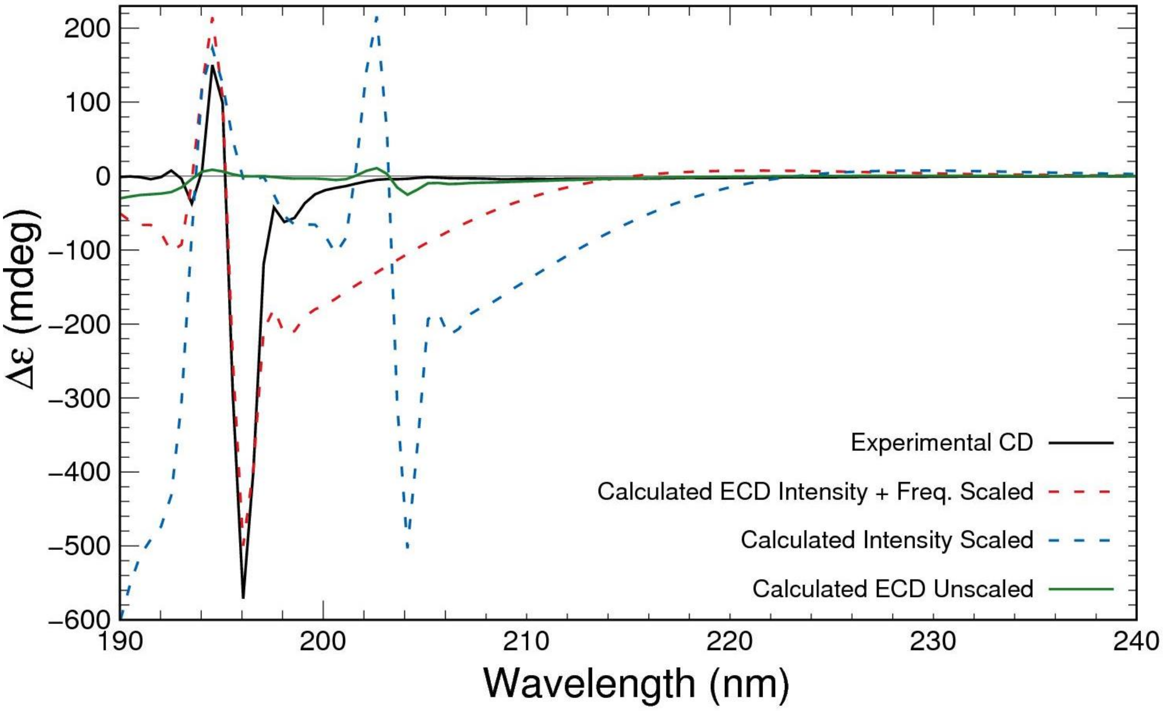

Figure S24 – Relative energies of all computed conformers of costatone C (**7**).

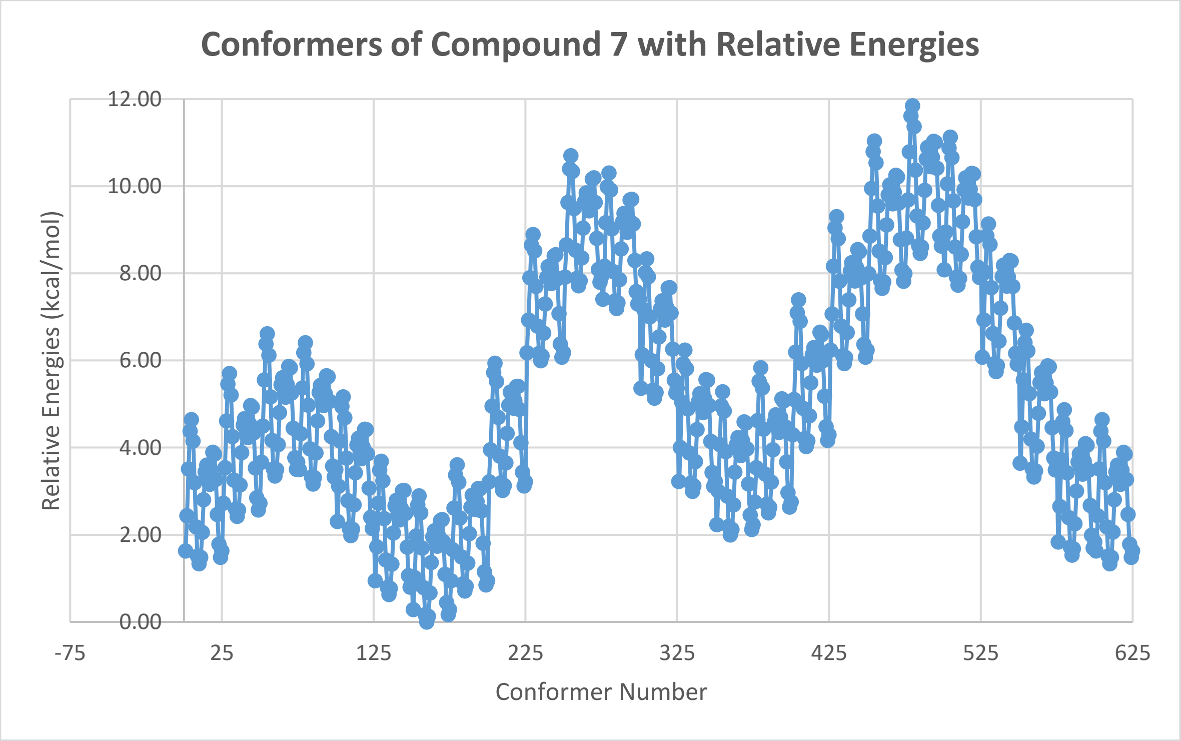

Supplement: Supplementary file 1 [file marinedrugs-17-00418-s001.pdf]
